# Effects of perioperative dexmedetomidine infusion on renal function and microcirculation in kidney transplant recipients: a randomised controlled trial

**DOI:** 10.1080/07853890.2022.2067351

**Published:** 2022-04-29

**Authors:** Yin-Chin Wang, Ming-Jiuh Wang, Chih-Yuan Lee, Chien-Chia Chen, Ching-Tang Chiu, Anne Chao, Wing-Sum Chan, Meng-Kun Tsai, Yu-Chang Yeh

**Affiliations:** a Department of Anesthesiology, National Taiwan University Hospital, Taipei, Taiwan; b Department of Surgery, National Taiwan University Hospital, Taipei, Taiwan; c Department of Anesthesiology, Far Eastern Memorial Hospital, New Taipei, Taiwan; d Department of Surgery, National Taiwan University Hospital, Hsin-Chu Branch, Hsinchu City, Taiwan

**Keywords:** Kidney, transplant, dexmedetomidine, microcirculation

## Abstract

**Objective:**

Ischemia-reperfusion injury affects postoperative transplanted kidney function in kidney transplant recipients. Dexmedetomidine was reported to attenuate ischemia-reperfusion injury and improve microcirculation, but its propensity to cause bradycardia and hypotension may adversely affect microcirculation. This study investigated the effect of dexmedetomidine on postoperative renal function and sublingual microcirculation in kidney recipients.

**Methods:**

The enrolled kidney transplant recipients were randomly allocated to the control group or dexmedetomidine group. After anaesthesia induction, patients in the dexmedetomidine group received dexmedetomidine infusion until 2 h after surgery. Sublingual microcirculation was recorded using an incident dark-field video microscope and analysed. The primary outcomes were the creatinine level on a postoperative day 2 and total vessel density at 2 h after surgery.

**Results:**

A total of 60 kidney recipients were analysed, and the creatinine levels on postoperative day 2 were significantly lower in the dexmedetomidine group than in the control group (1.5 (1.1–2.4) vs. 2.2 (1.7–3.0) mg/dL, median difference −0.6 (95% CI, −0.7 to −0.5) mg/dL, *p* = .018). On a postoperative day 7, the creatinine levels did not differ significantly between the two groups. Total vessel density at 2 h after surgery did not differ significantly between the two groups.

**Conclusion:**

We found that early postoperative renal function was better in kidney transplant recipients receiving dexmedetomidine infusion, but total vessel density was not significantly different between the intervention and control groups.

**Key messages**
- Ischemia-reperfusion injury affects postoperative transplanted kidney function, and dexmedetomidine was reported to attenuate ischemia-reperfusion injury and improve microcirculation in other clinical conditions.
- This study showed that early postoperative renal function was better in kidney transplant recipients receiving dexmedetomidine.
- Dexmedetomidine’s side effect of bradycardia and hypotension may affect microcirculation, our results revealed that the perioperative sublingual microcirculation did not differ significantly in kidney transplant recipients receiving dexmedetomidine.

## Introduction

End-stage kidney disease remains a global health concern; patients undergoing dialysis experience a lower quality of life and suffer increased morbidity and mortality. Kidney transplantation is the definitive treatment for patients on dialysis. However, ischemia-reperfusion injury to the transplanted kidneys may affect their postoperative function after kidney transplantation [1,2]. Moreover, renal microcirculation is a key issue related to acute and chronic kidney diseases [3]. Renal microvascular dysfunction includes alterations in endothelial barrier permeability, exaggerated inflammation, and impairment of endothelium-dependent vasorelaxation [3]. Our previous study revealed the alteration of sublingual microcirculatory dysfunction in patients on dialysis, and the severity of this alteration was lower in patients who receive a kidney transplant [4]. Surgical stress may affect the pre-existing microcirculatory dysfunction of patients undergoing kidney transplantation.

Dexmedetomidine is a highly selective alpha-2 agonist with sedative and analgesic effects [5]. It modulates inflammation by enhancing parasympathetic tone while reducing sympathetic tone [6,7]. Dexmedetomidine has been reported to confer renal protection effects in patients undergoing coronary artery bypass surgery [8,9]. The protective effects of dexmedetomidine against ischemia-reperfusion injury have been described in many studies [10–12]. Moreover, the sympatholysis effect of dexmedetomidine induced vasodilation [5], and our previous animal study showed that dexmedetomidine prevented alterations of intestinal microcirculation in rats with surgical stress and pain [13]. However, the most common side effects of dexmedetomidine are bradycardia and hypotension. Low cardiac output and low perfusion pressure may deteriorate microcirculation [14,15]. We hypothesised that dexmedetomidine could attenuate the ischemia-reperfusion injuries and preserve transplanted kidneys’ function. In addition, the issue that the effects of dexmedetomidine on perioperative microcirculation were protective or detrimental remained unknown. Thus, this study investigated postoperative renal function and perioperative sublingual microcirculation in patients undergoing kidney transplantation.

## Methods

### Patients

This prospective, randomised, controlled, single-blinded, open-label study was approved by the Research Ethics Committee of National Taiwan University Hospital, Taipei, Taiwan (Ethical Committee number 201512039MINB). This study was registered on the ClinicalTrials.gov protocol registration system (ID: NCT02707809). Patients undergoing kidney transplants were evaluated for eligibility. We excluded patients younger than 20 years or older than 70 years, and those with an allergy to dexmedetomidine, refractory bradycardia (heart rate below 60 beats per minute after treatment), and severe atrioventricular block (Mobitz type II and III). Written informed consent was obtained from all participants. The study was conducted and reported in accordance with the CONSORT recommendations [16]. The histidine-tryptophan-ketoglutarate solution was used for flushing and perfusion of the donor kidney, and the donor's kidney was kept in static cold storage before transplant surgery.

### Anaesthesia protocol and dexmedetomidine infusion protocol

The patients were allocated to a control group or dexmedetomidine group through randomised allocation using sealed opaque envelopes. The patients underwent sublingual microcirculation measurement before the induction of anaesthesia (T1). From before anaesthesia induction until 2 h after surgery, a non-invasive cardiac output monitoring system (NICOM, Cheetah Medical, Newton Centre, MA, USA) was used for measuring cardiac index, stroke volume index, and stroke volume variation. General anaesthesia was induced with fentanyl 1.5–2.0 µg/kg, propofol 1.5–3.0 mg/kg, glycopyrrolate 0.2 mg and cisatracurium 0.15–0.20 mg/kg and maintained with desflurane at an end-tidal concentration 4.2%–7.2%. Ventilator settings were as follows: fraction of inspired oxygen 40%; tidal volume 6–8 mL/kg; positive end-expiratory pressure level, 5 cm H_2_O; and end-tidal carbon dioxide partial pressure 35–40 mm Hg.

After anaesthesia induction, patients in the dexmedetomidine group received dexmedetomidine infusion until 2 h after surgery. To ensure patient safety, the titration rate of dexmedetomidine infusion was separately determined for each patient, and the infusion rate was adjusted in a range of 0.1–0.7 µg kg^−1^ *h*^−1^ according to the individual patient’s responses in terms of cardiac index, blood pressure, and heart rate. Fluid supplementation (in the range of 30–40 ml kg^−1^ of predicted body weight), ephedrine, and norepinephrine were used to maintain appropriate mean arterial pressure (MAP 70–100 mm Hg), heart rate (>60 beats per minute), cardiac index (>2.5 L/min/m^2^), and stroke volume variation (<13%). Patients undergoing ABO-incompatible kidney transplantation received 6 units of fresh frozen plasma during the operation. Patients in the control group received conventional management with fluid supplementation (30–40 mL/kg of predicted body weight), ephedrine, and norepinephrine to meet the same hemodynamic goal. During operation, the patients received methylprednisolone (10 mg/kg) for immunosuppression. At the end of the surgery, intravenous morphine 0.1–0.2 mg/kg was administered for postoperative analgesia. Extubation was performed in the operation room, and the patients were transferred to the post-anaesthesia care unit. After arrival at the post-anaesthesia care unit, the dexmedetomidine infusion rate was reduced by one-quarter of the initial rate every 30 min and discontinued after 2 h. After blood sample and microcirculation examinations were conducted, the patients were discharged from the post-anaesthesia care unit. In the general ward, postoperative fluid was administered according to the routine post kidney transplant care protocol, adjusted according to urine output and creatinine level. Postoperative creatinine level was measured once daily in the early morning during the follow-up period.

### Preoperative desensitisation and immunosuppressive therapy for ABO-incompatible kidney transplantation and postoperative immunosuppressive therapy

For ABO-incompatible kidney transplantation, preoperative desensitisation with rituximab and double filtration plasmapheresis and preoperative immunosuppressive therapy with tacrolimus, mycophenolate mofetil, and methylprednisolone were performed according to our published protocol [17]. Postoperative immunosuppressive therapy with tacrolimus, mycophenolate mofetil, and methylprednisolone was administered according to the post kidney transplant care protocol [17].

### Microcirculation examinations

Sublingual microcirculation images were captured using an incident dark-field video microscope (CytoCam, Braedius Medical, Huizen, the Netherlands). At each time point, six video sequences (length: 6 s each) were recorded at different sublingual sites, and three sequences with appropriate image quality were selected for analysis by a single observer who was blinded to the grouping. The analysis was performed using the semi-automated analysis software package Automated Vascular Analysis 3.0 (AVA, Academic Medical Centre, University of Amsterdam, Amsterdam, the Netherlands). Total vessel density (TVD, length of small vessels [less than 20 μm] in a 1-mm^2^ area [mm/mm^2^]), perfused vessel density (PVD, length of perfused small vessels in a 1-mm^2^ area [mm/mm^2^]), the proportion of perfused small vessels (PPV, PVD divided by TVD), and microvascular flow index (MFI, average of predominant flow classification [0–3] in four quadrants) were calculated according to round table conference guidelines [18,19].

### Timing of microcirculation examination, blood sample collection, and laboratory data recording

In addition to T1, perioperative microcirculation was examined at eight other time points in this study: T2, 1 h after anaesthesia induction; T3, 2 h after anaesthesia induction; T4, after ureterovesical anastomosis; T5, the end of surgery; T6, 2 h after surgery; POD1, postoperative day 1; POD2, postoperative day 2; and POD7, postoperative day 7. Blood samples were collected at T1, POD1, and POD2 to measure endocan, diamine oxidase, and neutrophil gelatinase-associated lipocalin (NGAL) levels. Urine samples were collected at T6, POD 1, and POD 2 for measuring urine NGAL levels. The blood urea nitrogen and creatinine levels on the day before surgery and at T6, POD1, POD2, POD3, and POD7 were recorded. Arterial blood gas analysis, electrolyte, and lactate levels were measured at T1, T3, T4, T5, and T6.

### Primary outcomes, sample size analysis, and other exploratory variables

The first primary outcome of this study was the difference in serum creatinine levels on postoperative day 2 between the two groups. According to our preliminary data, allocating 30 participants to each group provide sufficient power to detect a mean difference of 0.75 mg/dL in creatinine level between the two groups, with an *α* level of 0.05 (two-tailed) and a *β* level of 0.2 (80% power), assuming a controlled mean creatinine level of 2.5 mg/dL with a standard deviation of 1.0. The other primary outcome of this study was the difference in TVD at T6 between the two groups. According to our previous study [4], allocating 30 participants to each group provide sufficient power to detect a mean difference of 2.1 mm/mm^2^ in TVD between the two groups, with an *α* level of 0.05 (two-tailed) and a *β* level of 0.2 (80% power), assuming a controlled mean TVD of 22.8 mm/mm^2^ with a standard deviation of 2.7. Other exploratory variables included differences between the two groups in terms of hemodynamic variables; other microcirculation variables; lactate, blood urea nitrogen, creatinine, endocan, diamine oxidase, serum NGAL, and urine NGAL levels; tacrolimus level; and daily urine output at different time points.

### Randomisation and blinding methods

Block randomisation with a block size of 10 was used to randomise patients into the two groups and ensure a balance in sample size across the groups over time. Randomised allocation was performed by a research nurse in another research team in our department who was not otherwise involved in this study. She placed the sheet with the computerised random numbers generated from Excel software into sealed, opaque envelopes. The research nurse opened the sealed envelope before the induction of anaesthesia and confirmed the randomised number (0 = control, 1 = treatment, filled the checkbox in a case report from) and co-signed on the envelope with a colleague in the operation room. The envelope and randomised numbers were kept with the patient’s consent for reference. The video sequences of microcirculation were numbered, and the single observer who examined the microcirculation was blinded to the grouping.

### Statistical analysis

All data were analysed using SPSS 20 (IBM SPSS, Chicago, IL, USA). Normality was examined using the Shapiro-Wilk test. Normally distributed data were presented as means (standard deviation) and analysed using *t* test. Non–normally distributed data were presented as medians (interquartile range) and analysed using Mann-Whitney *U* test. Categorical data were analysed using a Fisher’s exact test. A *p* value > .05 was considered significant. The bootstrap method was used for calculating the median difference (95% confidence interval [CI]) for the comparisons of primary outcomes and lactate level.

## Results

### Patient characteristics, operation durations, and intraoperative medications

**Figure 1.**
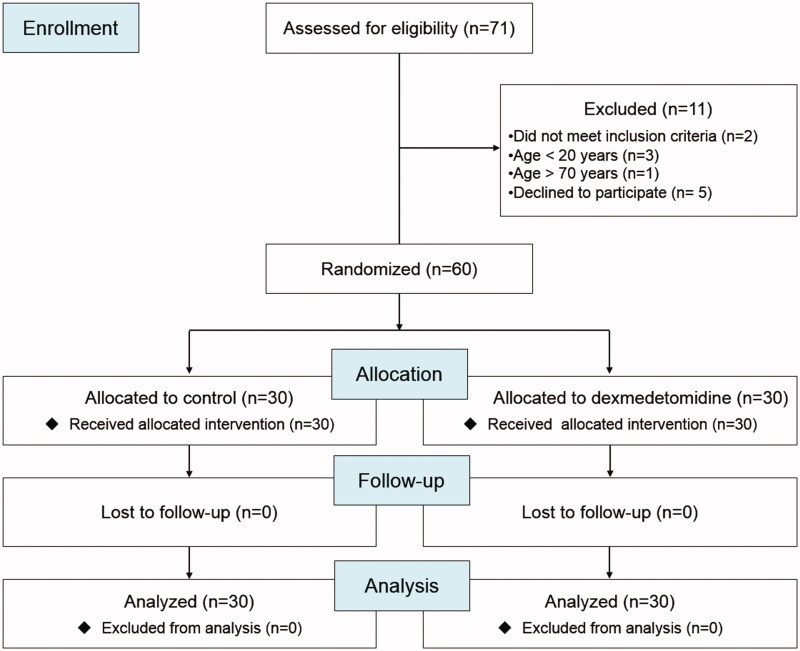
Consort flowchart of patient recruitment.

From August 2016 to March 2019, 71 patients undergoing kidney transplantation were assessed for eligibility, and 60 patients were enrolled (Figure 1). Patient characteristics, operation durations, and intraoperative medications are shown in Table 1. Fifty-eight patients received living kidney transplantation, and two patients in the control group received cadaveric kidney transplantation. The intraoperative infusion rate of dexmedetomidine was 0.19 (0.15–0.25) µg/kg/h for patients in the dexmedetomidine group. The end-tidal concentration of desflurane did not differ significantly between the two groups during the operation.

**Table 1.** Patient characteristics, operation duration, and perioperative management.

Group	Control	Dexmedetomidine
*n*	30	30
Female, *n* (%)	10 (33%)	12 (40%)
Age (years)	43 (34–53)	48 (27–56)
Weight (kg)	69.6 (14.5)	61.3 (10.7)
Height (cm)	168 (10)	166 (10)
Hemodialysis, *n* (%)	17 (57%)	19 (63%)
Peritoneal dialysis, *n* (%)	13 (43%)	11 (37%)
Medical history		
Hypertension, *n* (%)	16 (53%)	25 (83%)
Diabetes mellitus, *n* (%)	6 (20%)	4 (13%)
Coronary artery disease, *n* (%)	0 (0%)	5 (17%)
Preoperative BUN (mg/dL)	65 (29)	68 (25)
Preoperative creatinine (mg/dL)	11.5 (4.5)	11.3 (4.1)
ABO-incompatible transplantation	9 (30%)	8 (27%)
Operation duration (min)	196 (180–234)	217 (189–243)
Intraoperative management		
Dexmedetomidine (µg/kg/h)	–	0.19 (0.15–0.25)
Fluid supplement (mL)	2650 (2075–3000)	2250 (1775–2600)
Norepinephrine use, *n* (%)	19 (63%)	17 (57%)
Ephedrine use, *n* (%)	8 (27%)	10 (33%)
Furosemide use, *n* (%)	6 (20%)	6 (20%)
Postoperative 2 h at PACU		
Fluid supplement (mL)	300 (175–300)	300 (238–500)

Values are presented as number, number (%), means (standard deviation), medians (interquartile range). BUN, blood urea nitrogen; PACU, post-anaesthesia care unit.

### Primary outcomes

Creatinine levels at POD2 were significantly lower in the dexmedetomidine group than in the control group (1.5 (1.1–2.4) vs. 2.2 (1.7–3.0) mg/dL, median difference −0.6 (95% CI, −0.7 to −0.5) mg/dL, *p* = .018) (Table 2). After exclusion of the creatinine levels of the one patient with nephrectomy (6.6 mg/dL) in the dexmedetomidine group and two patients with a cadaveric kidney transplant (3.6 and 2.9 mg/dL, respectively) in the control group. The creatinine levels at POD 2 remained significantly lower in the dexmedetomidine group than in the control group (1.5 (1.1–2.4) vs 2.2 (1.6–2.7) mg/dL, median difference −0.6 (95% CI, −0.7 to −0.5) mg/dL, *p* = .016). TVD at T6 did not differ significantly between the dexmedetomidine and control groups (24.0 (22.8–24.9) vs. 24.1 (22.5–25.1) mm/mm^2^, median difference −0.1 (95% CI −0.2–0.1) mm/mm^2^, *p* = .918).

**Table 2.** Laboratory data, urine output, and organ injury markers.

Group	Control	Dexmedetomidine	*P* values
*N*	30	30	
Lactate (mmol/L)			
After induction of anaesthesia	0.9 (0.8–1.2)	0.8 (0.7–1.0)	.157
T3	1.3 (1.0–1.7)	0.9 (0.8–1.3)	.091
T4	1.2 (1.0–1.6)	1.0 (0.7–1.3)	.032
T5	1.5 (1.0–1.7)	1.0 (0.8–1.3)	.007
T6	1.4 (1.0–1.8)	1.0 (0.7–1.3)	.003
Creatinine (mg/dL)			
Postoperative day 1	5.4 (4.2–6.9)	3.8 (2.7–5.5)	.050
Postoperative day 2	2.2 (1.7–3.0)	1.5 (1.1–2.4)	.018
Postoperative day 3	1.6 (1.3–2.1)	1.3 (0.9–1.7)	.024
Postoperative day 7	1.3 (1.1–1.6)	1.1 (0.9–1.5)	.278
Blood urea nitrogen (mg/dL)			
Postoperative day 1	49.3 (35.6–60.2)	40.1 (26.3–51.0)	.061
Postoperative day 2	33.2 (24.4–43.1)	21.7 (15.7–32.9)	.008
Postoperative day 3	28.1 (23.6–40.3)	21.7 (17.8–32.2)	.034
Postoperative day 7	31.5 (23.9–42.3)	31.8 (26.7–35.8)	.796
Urine output (mL)			
Postoperative day 1	5035 (3581–8700)	7005 (3558–9795)	.246
Postoperative day 2	3740 (2784–5141)	4355 (3105–7743)	.196
Postoperative day 3	3640 (2758–4655)	3915 (3070–5465)	.304
Serum NGAL (ng/mL)			
After induction of anaesthesia	1854 (915–4119)	1444 (649–3986)	.409
Postoperative day 1	2161 (375–7963)	2086 (682–4679)	.666
Postoperative day 2	931 (482–5210)	672 (517–1968)	.459
Urine NGAL (ng/mL)			
T6	528 (247–855)	420 (300–1139)	.486
Postoperative day 1	184 (71–425)	171 (59–493)	.877
Postoperative day 2	141 (77–308)	98 (3–262)	.299
Endocan (ng/mL)			
After induction of anaesthesia	0.91 (0.48–1.60)	0.99 (0.62–2.99)	.824
Postoperative day 1	0.78 (0.45–1.59)	0.93 (0.55–1.59)	.932
Postoperative day 2	0.79 (0.43–1.54)	0.91 (0.62–2.15)	.265
Diamine oxidase (U/L)			
After induction of anaesthesia	2.6 (1.0–5.9)	3.3 (1.7–7.5)	.165
Postoperative day 1	5.5 (2.6–10.3)	6.1 (1.4–11.2)	.744
Postoperative day 2	4.5 (2.3–8.6)	5.9 (3.8–8.3)	.447

Values are median (interquartile range). NGAL, neutrophil gelatinase-associated lipocalin; T3, 2 h after anaesthesia induction; T4, after ureterovesical anastomosis; T5, the end of surgery; T6, 2 h after surgery.

### Other exploratory variables

#### Macrocirculation variables

**Figure 2.**
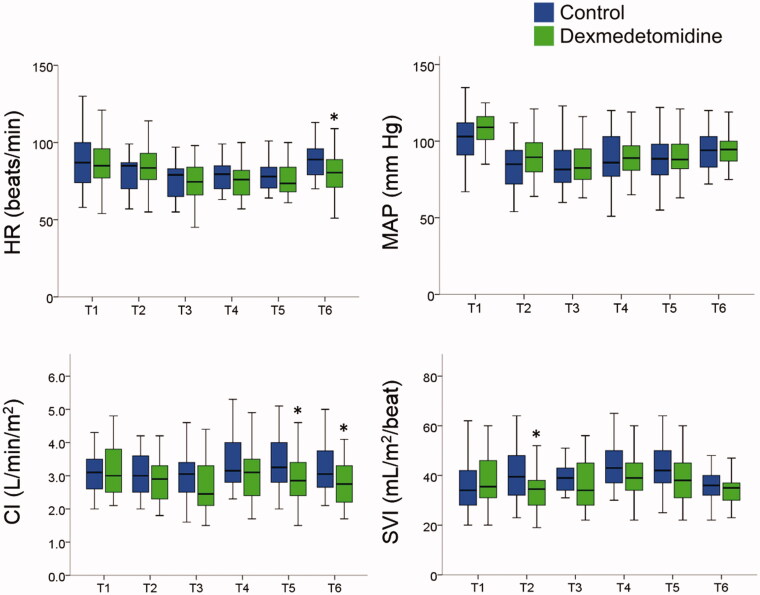
Hemodynamic variables at each time point. CI, cardiac index; HR, heart rate; MAP, mean arterial pressure; SVI, stroke volume index. *P < .05 indicates significant differences between the two groups determined using the Mann-Whitney *U* test. T1, before anaesthesia induction; T2, 1 h after anaesthesia induction; T3, 2h after anaesthesia induction; T4, after ureterovesical anastomosis; T5, the end of surgery; T6, 2h after surgery.

Hemodynamic variables are shown in Figure 2. Heart rate at T6 was lower in the dexmedetomidine group than in the control group (81 (71–89) vs. 89 (79–96) beats per minute, *p* = .015). MAP did not differ significantly between the two groups. Cardiac indexes at T5 and T6 were lower in the dexmedetomidine group than in the control group (T5, 2.9 (2.4–3.5) vs. 3.3 (2.8–4.0) L/min/m^2^, *p* = .017; T6, 2.8 (2.2–3.3) vs. 3.1 (2.7–3.8) L/min/m^2^, *p* = .04]. Stroke volume index at T2 was lower in the dexmedetomidine group than in the control group (35 (28–39) vs. 40 (32–49) mL/m^2^/beat, *p* = .035). Daily urine output did not differ significantly between the two groups at each time point (Table 2).

#### Microcirculatory variables

**Figure 3.**
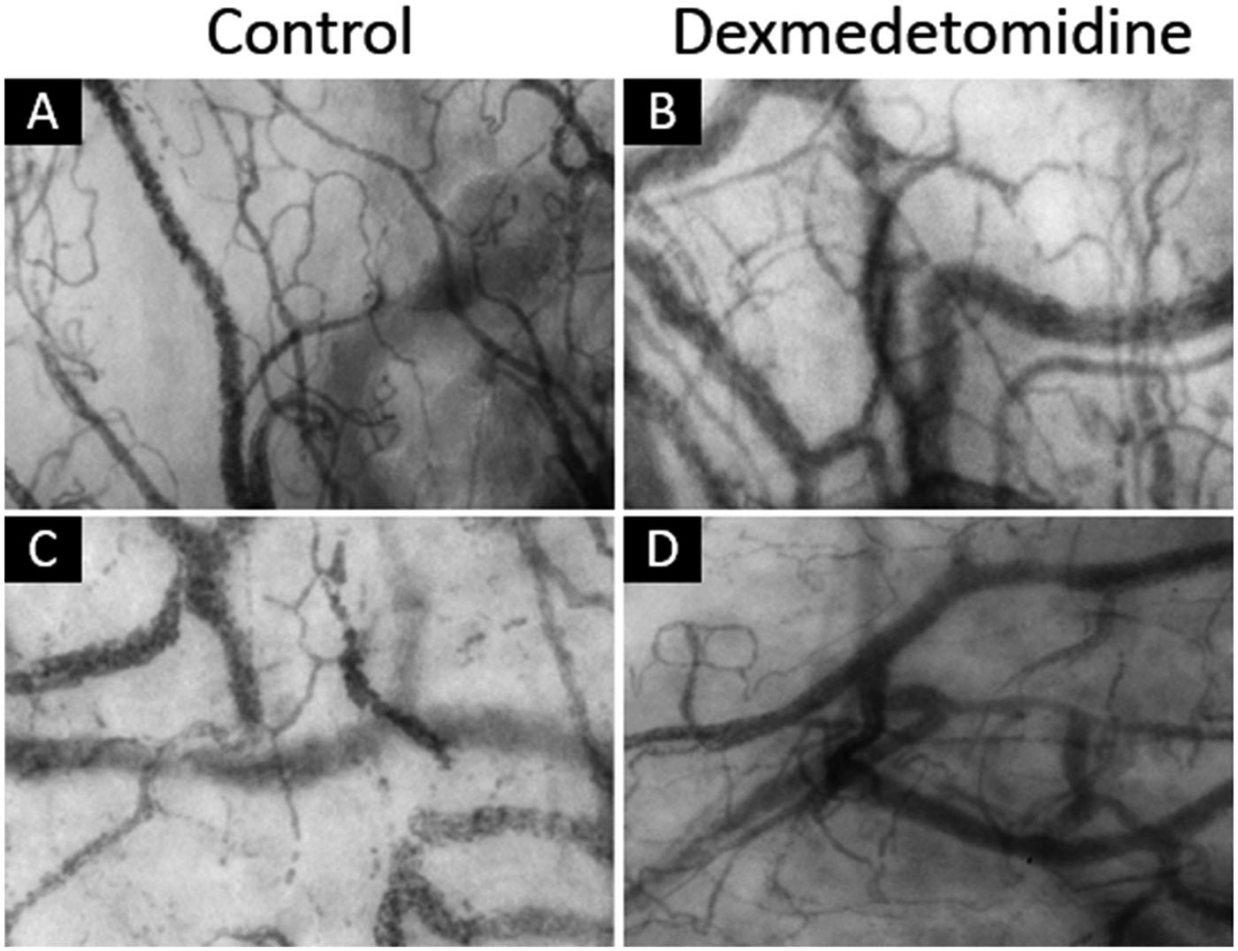
Sublingual microcirculation images at 2 h after surgery. (A and B) represent good microcirculation in one control group patient (PVD: 25.9 mm/mm^2^) and one dexmedetomidine (Dex) group patient (PVD: 25.8 mm/mm^2^). (C and D) represent fewer perfused vessels in one control group patient (PVD: 22.7 mm/mm^2^) and one dexmedetomidine group patient (PVD: 22.8 mm/mm^2^). PVD, perfused vessel density.

**Figure 4.**
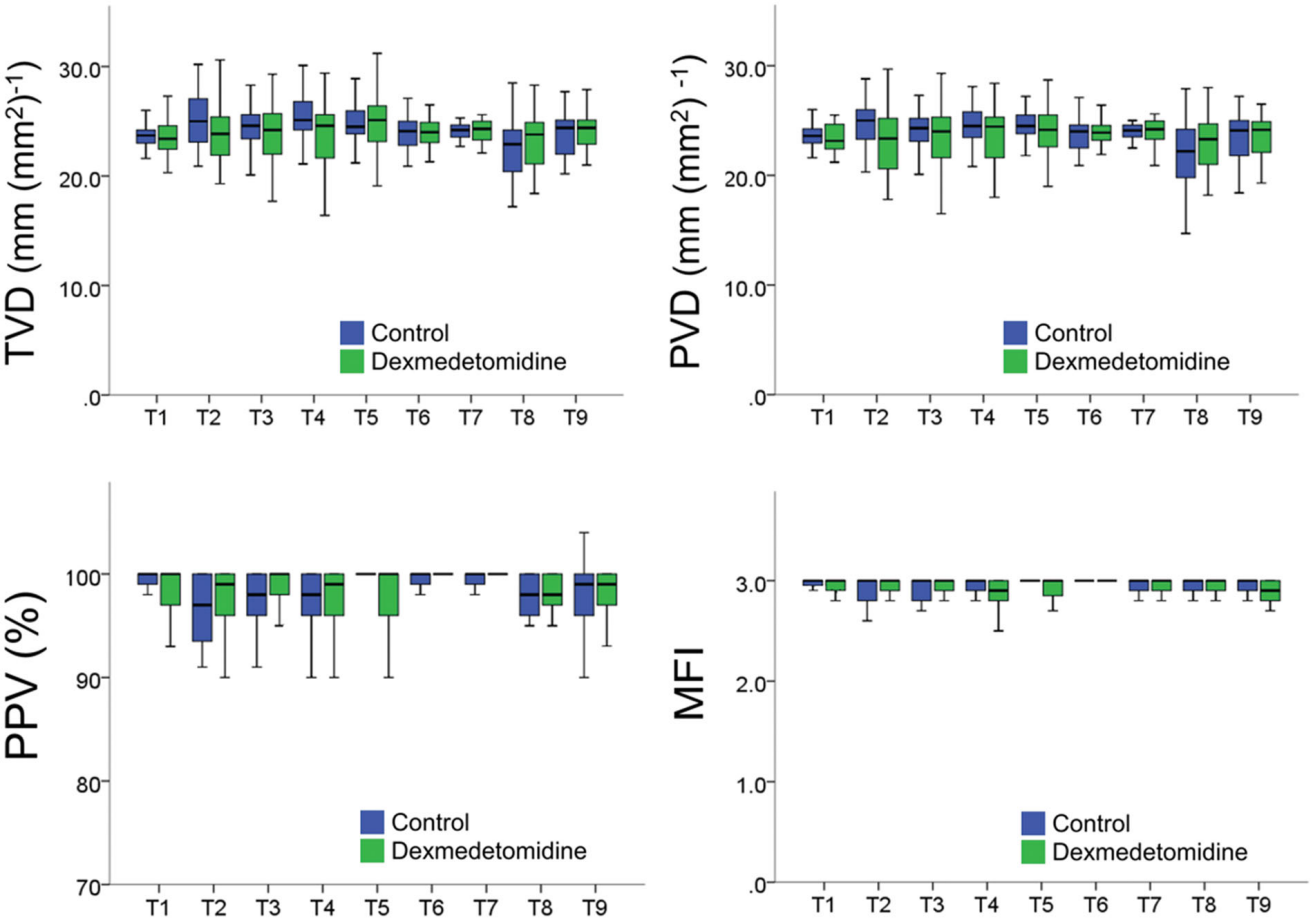
Microcirculation variables at each time point. Microcirculation variables did not differ significantly between the control and dexmedetomidine (Dex) groups. The definitions of time points are as follows. T1, before anaesthesia induction; T2, 1 h after anaesthesia induction; T3, 2h after anaesthesia induction; T4, after ureterovesical anastomosis; T5, the end of surgery; T6, 2h after surgery; T7, postoperative day 1; T8, postoperative day 2; T9, postoperative day 7. MFI, microvascular flow index; PPV, proportion of perfused vessels; PVD, perfused vessel density; TVD, total vessel density.

Images depicting the sublingual microcirculation of several patients at 2 h after surgery (T6) are shown in Figure 3. TVD and PVD did not differ significantly between the two groups (Figure 4). PPV and MFI did not differ significantly between the two groups.

#### Laboratory data variables

In addition to POD2, creatinine levels were higher in the control group than in the dexmedetomidine group at POD1 and POD3 (Table 2). Blood urea nitrogen levels were higher in the control group than in the dexmedetomidine group at POD2 and POD3. Lactate levels were significantly lower in the dexmedetomidine group than in the control group at T4, T5, and T6 [T6, 1.0 (0.7–1.3) vs. 1.4 (1.0–1.8) mmol/L, median difference −0.4 (95% CI −0.4 to −0.3) mmol/L, *p* = .003]. Other laboratory data variables, including endocan, diamine oxidase, serum NGAL and urine NGAL levels did not differ significantly between the two groups. Tacrolimus levels at POD3 and POD7 did not differ significantly between the control and dexmedetomidine groups (POD3, 7.6 (4.9–11.5) vs. 6.7 (5.1–10.3) ng/mL, *p* = .631; POD7, 6.5 (5.0–8.5) vs. 7.0 (5.8–8.5) ng/mL, *p* = .610).

### Post-hoc analysis

During the manuscript reviewing process, a post-hoc analysis was requested. Three patients were excluded from the post-hoc analysis as follows: two patients in the control group received cadaveric kidney transplantation and one patient in the dexmedetomidine group underwent nephrectomy of the transplanted kidney 10 days after the operation for acute rejection and infarction of the transplanted kidney. After exclusion of the three patients, the patient characteristics, operation durations, and intraoperative medications are shown in Supplementary Table 1. The creatinine levels on POD 2 remained significantly lower in the dexmedetomidine group than in the control group (1.5 (1.1–2.4) vs 2.2 (1.6–2.7) mg/dL, median difference −0.6 (95% CI, −0.7 to −0.5) mg/dL, *p* = .016). TVD at T6 did not differ significantly between the dexmedetomidine and control groups (24.1 (22.9–24.6) vs. 24.0 (22.9–25.1) mm/mm^2^, median difference 0.1 (95% CI, −0.1–0.3) mm/mm^2^, *p* = .804). Hemodynamic variables are shown in Supplementary Figure 1. TVD and PVD did not differ significantly between the two groups (Supplementary Figure 2). PPV and MFI did not differ significantly between the two groups.

In addition to POD2, creatinine levels were higher in the control group than in the dexmedetomidine group at POD1 and POD3 (Supplementary Table 2). Blood urea nitrogen levels were higher in the control group than in the dexmedetomidine group on POD2 and POD3. Lactate levels were significantly lower in the dexmedetomidine group than in the control group at T6 (.0 (0.7–1.4) vs. 1.3 (1.0–1.9) mmol/L, median difference −0.3 (95% CI, −0.4 to −0.3) mmol/L, *p* = .008).

## Discussion

In this study, we observed that creatinine and blood urea nitrogen levels on POD2 were lower in the dexmedetomidine group than in the control group. However, the creatinine level on POD7 did not differ significantly between the two groups. Moreover, sublingual microcirculation did not differ significantly between the two groups at 2 h after surgery.

Several potential mechanisms may explain the finding that early postoperative renal function after dexmedetomidine infusion was better in this study. First, several studies have reported that dexmedetomidine improved regional perfusion [13,20,21]. The favourable effect of such perfusion on mesenteric microcirculation had been reported in several studies [13,20]. Second, dexmedetomidine has been reported to attenuate inflammation and reduce ischemic–reperfusion injury in several studies [22–24]. However, we did not observe significant differences in the kidney and intestinal injury markers between the two groups. The intraoperative administration of high-dose steroids for immunosuppression in kidney transplant recipients may have attenuated the difference in inflammation and ischemia-reperfusion injury between the two groups. Third, dexmedetomidine’s effect on polyuria has been reported in several studies [8,25]. Because this study did not aim to investigate differences in daily urine output, our non-significantly higher daily urine output at POD1 and POD2 after dexmedetomidine treatment may suggest that further studies are warranted to investigate dexmedetomidine’s effect on urine output after kidney transplantation. In addition, a retrospective cohort study reported that perioperative dexmedetomidine uses the reduced incidence of delayed graft function, risk of infection, risk of acute rejection, overall complication, and length of hospital stay [26].

Two factors may explain how microcirculation could be preserved after dexmedetomidine treatment at 2 h after surgery. First, to ensure patient safety, we did not apply a fixed dexmedetomidine infusion rate during the operation; we used blood pressure, heart rate, and cardiac index to determine the dexmedetomidine infusion rate for each patient. Mohamed *et al.* reported that high dexmedetomidine infusion (0.5 µg kg^−1^ *h*^−1^) improved sublingual microcirculation variables in patients undergoing on-pump coronary artery bypass graft surgery [27]. Second, it has been reported that increased MAP improves microcirculation and reduces the incidence of renal failure in patients with sepsis [28,29]. In our previous study, we found that MAP was moderately positively correlated to microcirculation in patients on dialysis [4]. Because of adequate fluid supplements and medications, the median MAP of both groups in our study exceeded 80 mm Hg from T1 to T6.

Although a concomitant analysis of sublingual microcirculation mirrored the findings of a contrast-enhanced ultrasound examination of the kidney in a septic animal study [30], the correlation between sublingual microcirculation and microcirculation of transplanted kidney remains unknown. Please notice that our results of sublingual microcirculation could not directly reflect the change in the microcirculation of the transplanted kidney. Further studies are warranted to apply other advanced imaging techniques to investigate the microcirculation on the surface or inside of the transplanted kidney. These include a full-field laser perfusion imager by employing the laser speckle contrast imaging technique [21], contrast-enhanced ultrasound [31], and magnetic resonance imaging technique [32].

Preoperative desensitisation and immunosuppressive therapy in ABO-incompatible kidney recipients might attenuate the anti-inflammatory effect of dexmedetomidine during the operation. The randomisation design of this study prevented the significantly unequal number of patients with ABO-incompatible transplantation between the two groups. Moreover, tacrolimus was used in this study, and it had the potential to affect renal function. We did not observe that tacrolimus levels were significantly different between the two groups.

## Limitations

This study has several limitations. First, this was a single-centre study. Different protocols of fluid supplementation, target MAP, and cardiac output goal may have different influences on the effects of dexmedetomidine on renal function and microcirculation. Many concurrent interventions during kidney transplants make it difficult to identify a mechanism for the observed effects. Second, the use of dexmedetomidine was not blinded. There were two reasons for not blinding. One was the safety issue of living kidney transplantation, and we aimed to maintain adequate cardiac output for all participants. The other was the difficulty to blind the bradycardia effect of dexmedetomidine. Third, the sample size of this study was not designed and powered to detect the difference between the two groups on POD7. Further studies are required to investigate the longer effect of dexmedetomidine. Fourth, two patients with cadaver kidney transplants in the control group were excluded from the analysis, their creatinine levels on postoperative day 2 were 3.6 and 2.9 mg/dL respectively. Further study is required to investigate the effect of dexmedetomidine in patients with cadaver kidney transplantation. Fifth, there are many components to performing the postoperative bundle care of kidney transplant recipients, and further studies are required to investigate other interventions after kidney transplantation for early recovery or long-term preservation of renal function.

## Conclusions

In conclusion, we found that early postoperative renal function was better in kidney transplant recipients receiving perioperative dexmedetomidine infusion. The sublingual microcirculation in kidney transplant recipients of the dexmedetomidine group was preserved by maintaining adequate cardiac output and mean arterial pressure. Further studies are warranted to investigate the mechanism and effects of dexmedetomidine on other postoperative clinical outcomes in kidney transplant recipients.

## Supplementary Material

Supplemental Material

## Data Availability

The data that support the findings of this study are available from the corresponding author upon reasonable request.
