# Supplementary material for: Effects of perioperative dexmedetomidine infusion on renal function and microcirculation in kidney transplant recipients: a randomised controlled trial
Source: Ann Med. 2022 Apr 29;54(1):1233–43. doi: 10.1080/07853890.2022.2067351 (PMC9126588; doi:10.1080/07853890.2022.2067351)
Supplement: Supplemental Material [file IANN_A_2067351_SM9554.docx]

**Effects of perioperative dexmedetomidine infusion on renal function and microcirculation in kidney transplant recipients: a randomized controlled trial**

Yin-Chin Wang, Ming-Jiuh Wang, Chih-Yuan Lee, Chien-Chia Chen, Ching-Tang Chiu, Anne Chao, Wing-Sum Chan, Meng-Kun Tsai, Yu-Chang Yeh

**Supplementary File**

Supplementary Figure 1 ............................... 1

Supplementary Figure 2 ............................... 2

Supplementary Table 1 ................................ 3

Supplementary Table 2 ................................ 4

**Supplementary Figure 1.** Hemodynamic variables at each time point.

Three patients were excluded from the post-hoc analysis as follows: two patients in the control group received cadaveric kidney transplantation and one patient in the dexmedetomidine group underwent nephrectomy of the transplanted kidney 10 days after operation for acute rejection and infarction of the transplanted kidney. CI, cardiac index; HR, heart rate; MAP, mean arterial pressure; SVI, stroke volume index. **P*<0.05 indicates significant differences between the two groups determined using the Mann–Whitney U test. T1, before anesthesia induction; T2, 1 h after anesthesia induction; T3, 2h after anesthesia induction; T4, after ureterovesical anastomosis; T5, the end of surgery; T6, 2h after surgery.


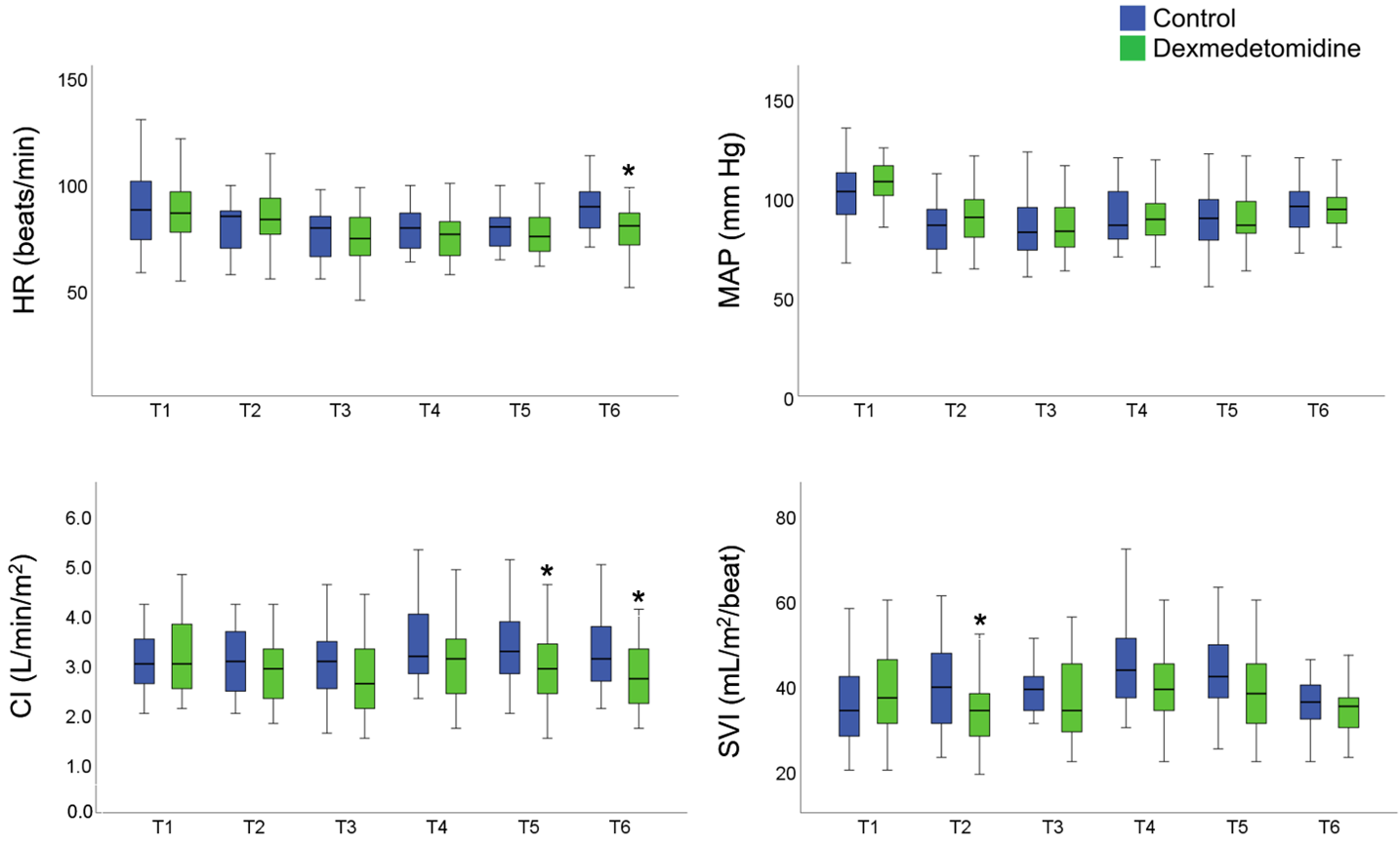


**Supplementary Figure 2.** Microcirculation variables at each time point.

Microcirculation variables did not differ significantly between the control and dexmedetomidine (Dex) groups. Three patients were excluded from the post-hoc analysis as follows: two patients in the control group received cadaveric kidney transplantation and one patient in the dexmedetomidine group underwent nephrectomy of the transplanted kidney 10 days after operation for acute rejection and infarction of the transplanted kidney. The definitions of time points are as follows. T1, before anesthesia induction; T2, 1 h after anesthesia induction; T3, 2h after anesthesia induction; T4, after ureterovesical anastomosis; T5, the end of surgery; T6, 2h after surgery; T7, postoperative day 1; T8, postoperative day 2; T9, postoperative day 7. MFI, microvascular flow index; PPV, proportion of perfused vessels; PVD, perfused vessel density; TVD, total vessel density.


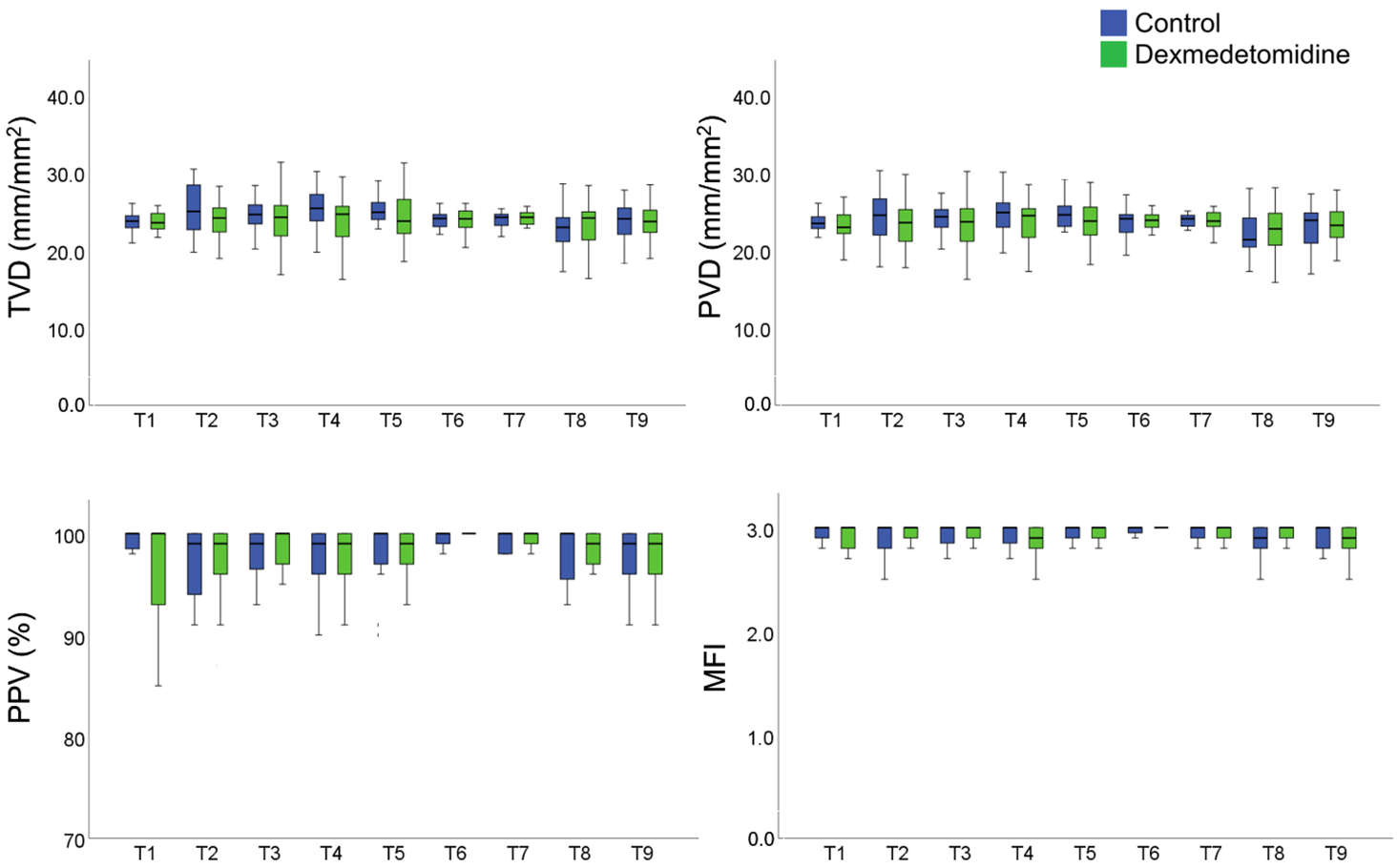


**Supplementary Table 1** Patient characteristics, operation duration, and perioperative management

| **Group** | **Control** | **Dexmedetomidine** |
| --- | --- | --- |
| *n* | 28 | 29 |
| Female, *n* (%) | 9 (32%) | 11 (38%) |
| Age (years) | 46 (35 to 54) | 48 (26 to 56) |
| Weight (kg) | 69.8 (60-77.6) | 58.6 (54.8-68) |
| Height (cm) | 169 (160-174) | 165 (158-175) |
| Hemodialysis, n (%) | 15 (54%) | 18 (62%) |
| Peritoneal dialysis, n (%) | 13 (46%) | 11 (38%) |
| Medical History |  |  |
| Hypertension, *n* (%) | 14 (50%) | 24 (83%) |
| Diabetes mellitus, *n* (%) | 6 (21%) | 4 (14%) |
| Coronary artery disease, *n* (%) | 0 (0%) | 5 (17%) |
| Preoperative BUN (mg/dL) | 58 (42-90) | 68 (51-85) |
| Preoperative creatinine (mg/dL) | 11.5 (7.4-16) | 10.9 (9.3-13.7) |
| ABO-incompatible transplantation | 9 (32%) | 8 (28%) |
| Operation duration (min) | 195 (180 to 218) | 216 (189 to 246) |
| **Intraoperative management** |  |  |
| Dexmedetomidine (µg/kg/h) | - | 0.19 (0.15 to 0.23) |
| Fluid supplement (mL) | 2200 (1800 to 2875) | 2200 (1275 to 2550) |
| Norepinephrine use, *n* (%) | 17 (61%) | 17 (59%) |
| Ephedrine use, *n* (%) | 7 (25%) | 10 (34%) |
| Furosemide use, *n* (%) | 5 (18%) | 5 (17%) |
| **Postoperative 2 h** at PACU |  |  |
| Fluid supplement (mL) | 300 (200 to 300) | 300 (225 to 475) |

Values are presented as number, number (%), means (standard deviation), medians (interquartile range). Three patients were excluded from the post-hoc analysis as follows: two patients in the control group received cadaveric kidney transplantation and one patient in the dexmedetomidine group underwent nephrectomy of the transplanted kidney 10 days after operation for acute rejection and infarction of the transplanted kidney. BUN, blood urea nitrogen; PACU, post-anesthesia care unit.

**Supplementary Table 2** Laboratory data, urine output, and organ injury markers

| **Group** | **Control** | **Dexmedetomidine** | *P* values |
| --- | --- | --- | --- |
| *n* | 28 | 29 |  |
| Lactate (mmol/L) |  |  |  |
| After induction of anesthesia | 0.9 (0.8 to 1.3) | 0.8 (0.7 to 1.1) | 0.077 |
| T3 | 1.3 (1.0 to 1.7) | 0.9 (0.8 to 1.3) | 0.040 |
| T4 | 1.3 (1.0 to 1.6) | 1.0 (0.7 to 1.3) | 0.031 |
| T5 | 1.5 (1.0 to 1.7) | 1.0 (0.8 to 1.3) | 0.011 |
| T6 | 1.3 (1.0 to 1.9) | 1.0 (0.7 to 1.4) | 0.008 |
| Creatinine (mg/dL) |  |  |  |
| Postoperative day 1 | 5.4 (4.1 to 7.0) | 3.8 (2.8 to 5.6) | 0.036 |
| Postoperative day 2 | 2.2 (1.6 to 2.7) | 1.5 (1.1 to 2.4) | 0.016 |
| Postoperative day 3 | 1.6 (1.2 to 2.1) | 1.3 (0.9 to 1.6) | 0.022 |
| Postoperative day 7 | 1.3 (1.0 to 1.6) | 1.1 (0.9 to 1.5) | 0.238 |
| Blood urea nitrogen (mg/dL) |  |  |  |
| Postoperative day 1 | 49.3 (34.5 to 60.3) | 38.2 (26.1 to 50.2) | 0.049 |
| Postoperative day 2 | 33.1 (23.8 to 42.5) | 21.1 (15.1 to 31.7) | 0.010 |
| Postoperative day 3 | 27.7 (23.2 to 39.4) | 21.7 (17.6 to 31.5) | 0.029 |
| Postoperative day 7 | 30.9 (23.8 to 39.8) | 31.7 (26.7 to 35.7) | 0.955 |
| Urine output (mL) |  |  |  |
| Postoperative day 1 | 5200 (3623 to 9360) | 7020 (3985 to 10340) | 0.250 |
| Postoperative day 2 | 4020 (2986 to 5153) | 4440 (3110 to 7955) | 0.183 |
| Postoperative day 3 | 3738 (3108 to 4845) | 3920 (3125 to 5720) | 0.367 |
| Serum NGAL (ng/mL) |  |  |  |
| After induction of anesthesia | 2035 (945 to 4521) | 1419 (637 to 4036) | 0.302 |
| Postoperative day 1 | 2903 (364 to 8000) | 2145 (747 to 5037) | 0.876 |
| Postoperative day 2 | 924 (539 to 5198) | 672 (517 to 2143) | 0.560 |
| Urine NGAL (ng/mL) |  |  |  |
| T6 | 352 (231 to 750) | 474 (299 to 1161) | 0.147 |
| Postoperative day 1 | 153 (71 to 442) | 136 (46 to 509) | 0.987 |
| Postoperative day 2 | 141 (49 to 322) | 102 (39 to 256) | 0.511 |
| Endocan (ng/mL) |  |  |  |
| After induction of anesthesia | 0.91 (0.65 to 1.60) | 0.99 (0.62 to 2.99) | 0.863 |
| Postoperative day 1 | 0.78 (0.45 to 1.59) | 0.93 (0.55 to 1.59) | 0.596 |
| Postoperative day 2 | 0.79 (0.43 to 1.54) | 0.91 (0.62 to 2.15) | 0.318 |
| Diamine oxidase (U/L) |  |  |  |
| After induction of anesthesia | 2.8 (1.1 to 6.6) | 3.8 (1.8 to 7.9) | 0.283 |
| Postoperative day 1 | 6.2 (2.7 to 10.5) | 6.1 (3.6 to 11.7) | 0.866 |
| Postoperative day 2 | 4.6 (2.7 to 9.0) | 6.1 (4.0 to 8.3) | 0.495 |

Values are median (interquartile range). Three patients were excluded from the post-hoc analysis as follows: two patients in the control group received cadaveric kidney transplantation and one patient in the dexmedetomidine group underwent nephrectomy of the transplanted kidney 10 days after operation for acute rejection and infarction of the transplanted kidney. NGAL, neutrophil gelatinase-associated lipocalin; T3, 2h after anesthesia induction; T4, after ureterovesical anastomosis; T5, the end of surgery; T6, 2h after surgery.
